# GRK2 promotes growth of medulloblastoma cells and protects them from chemotherapy-induced apoptosis

**DOI:** 10.1038/s41598-019-50157-5

**Published:** 2019-09-25

**Authors:** Anup S. Pathania, Xiuhai Ren, Min Y. Mahdi, Gregory M. Shackleford, Anat Erdreich-Epstein

**Affiliations:** 1 Department of Pediatrics, Division of Hematology, Oncology and Blood and Marrow Transplantation, The Saban Research Institute at Children’s Hospital Los Angeles and Keck School of Medicine, University of Southern California, Los Angeles, California USA; 2 Department of Pediatrics, Division of Hematology, Oncology and Blood and Marrow Transplantation, The Saban Research Institute at Children’s Hospital Los Angeles, Los Angeles, California USA; 3 Department of Radiology, The Saban Research Institute at Children’s Hospital Los Angeles, Los Angeles, California USA; 4 Department of Pediatrics, Division of Hematology, Oncology and Blood and Marrow Transplantation, The Saban Research Institute at Children’s Hospital Los Angeles and Keck School of Medicine and Norris Comprehensive Cancer Center, University of Southern California, Los Angeles, California USA; 5 Department of Pathology, Children’s Hospital Los Angeles and Keck School of Medicine, University of Southern California, Los Angeles, California USA

**Keywords:** Cancer, Cell biology

## Abstract

G-protein coupled receptor kinase 2 (GRK2; ADRBK1, BARK1) is most known as a regulator of G-protein coupled receptors. However, GRK2 also has other functions. Medulloblastomas are the most common malignant brain cancers in children. GRK2 has not been implicated in medulloblastoma biology. Here we report that GRK2 knockdown slowed cell growth, diminished proliferation, and enhanced cisplatin- and etoposide-induced apoptosis in medulloblastoma cell lines UW228-2 and Daoy. Reciprocally, GRK2 overexpression attenuated apoptosis induced by these chemotherapy drugs. Cisplatin and etoposide increased phosphorylation of AKT (S473) and GRK2 knockdown mitigated this increase. Cisplatin and etoposide attenuated ERK phosphorylation, but GRK2 knockdown did not alter this effect. Wildtype GRK2 reversed the increase in cisplatin- and etoposide-induced apoptosis caused by GRK2 knockdown. GRK2-K220R (kinase dead) and GRK2-S670A (unphosphorylated, constitutively active) conferred protection from cisplatin that was similar to wildtype GRK2, suggesting that this protection may be mediated though a kinase-independent activity of GRK2. These data demonstrate that GRK2 contributes to proliferation and survival of these medulloblastoma cell lines and to their protection from cisplatin- and etoposide-induced apoptosis.

## Introduction

G-protein coupled receptor kinase 2 (GRK2) belongs to the GRK family of serine/threonine kinases and is largely known as a regulator of G-protein coupled receptor (GPCR) signaling, including the β2 adrenergic receptor^1–5^. This is through phosphorylation of agonist-stimulated GPCRs, causing $\beta$-arrestin-mediated uncoupling from G-proteins, that in turn promotes clathrin-dependent GPCR internalization and recycling, thus regulating GPCR activity^6–12^. GRK2, through its regulatory effect on activity of the β2 adrenergic receptor, protects heart muscle from detrimental effects of prolonged adrenergic stimulation, and accordingly, inhibitors of GRK2 are in development for conditions involving heart failure^13–20^. GRK2 can also be recruited to GPCRs without any G-protein activation, as shown for the dopamine D2 receptor^21^. GRK2 functions in diverse molecular pathways and affects multiple cellular processes beyond regulating GPCR signaling^22,23^. For example, GRK2 can phosphorylate non-GPCR receptors and substrates such as platelet-derived growth factor receptor (PDGFR)-$\beta$^24^, tubulin^25^, insulin receptor substrate (IRS)-1^26^, Smad-2, Smad-3^27^, IκBα^28^, p38^29^, ezrin^30^ and histone deacetylase (HDAC) 6^31^. Other examples are inhibition of GPCR-independent C-C motif chemokine ligand (CCL)2-induced ERK phosphorylation^32^ and direct binding to AKT to inhibit its phosphorylation^33^. Thus, GRK2 is involved in multiple signaling pathways in diverse biological functions.

Medulloblastomas are embryonal tumors that, as a group, constitute the most common malignant brain tumors in children and are also seen in adults. Medulloblastomas are heterogenous in terms of their histology, biology and prognosis, with approximately 5% being associated with cancer predisposition syndromes^34^. Current molecular classification divides medulloblastomas into at least four molecular subgroups: WNT, Sonic Hedgehog (SHH), Group 3 and Group 4, initially defined based on expression microarrays and reflecting their different biology^35–40^. Most WNT medulloblastomas have activating/stabilizing somatic mutations in β-catenin and are characterized by increased WNT pathway signaling^35,40,41^. SHH medulloblastomas show unregulated ligand-independent SHH pathway signaling due to mutations/alterations such as loss-of-function of *PATCHED* (*PTCH1*) or Suppressor of Fused (*SUFU*), gain-of-function of Smoothened (*SMO*), *GLI1*, *GLI2*, or *MYCN*, and/or other alterations^41–43^. An especially poor-prognosis subgroup of SHH medulloblastomas are those with somatic or germline mutations in *TP53*^44^. Group 3 medulloblastomas are considered most aggressive of the four main subgroups overall, and have propensity for leptomeningeal metastases at diagnosis^36,45^. While amplification of *MYC* defines Group 3 medulloblastomas and confers poor prognosis, such *MYC* amplification occurs in less than a quarter of Group 3 tumors, with a heterogenous set of other molecular mechanisms occurring in the remainder^41,43^. Group 4 medulloblastomas lack a defining single somatic gene mutation, and like Group 3, are associated with a variety of molecular alterations and signaling pathways, some of which overlap with Group 3 tumors^37,41,43,46,47^. Advances in molecular analyses continue to further subdivide medulloblastoma subgroups taking advantage of newly-emerging knowledge and methodology^48,49^. GRK2 can regulate SHH/SMO signaling at different points along their signaling pathway^10,50–59^ and is expressed in medulloblastomas, but to date, has not been implicated in medulloblastoma biology.

In the present study we demonstrate for the first time a pro-survival role for GRK2 in two SHH subgroup medulloblastoma cell lines and GRK2-mediated mitigation of their cisplatin- and etoposide-induced apoptosis.

## Results

### GRK2 knockdown slows cell growth and diminishes proliferation of medulloblastoma cell lines UW228 and Daoy

**Figure 1:**
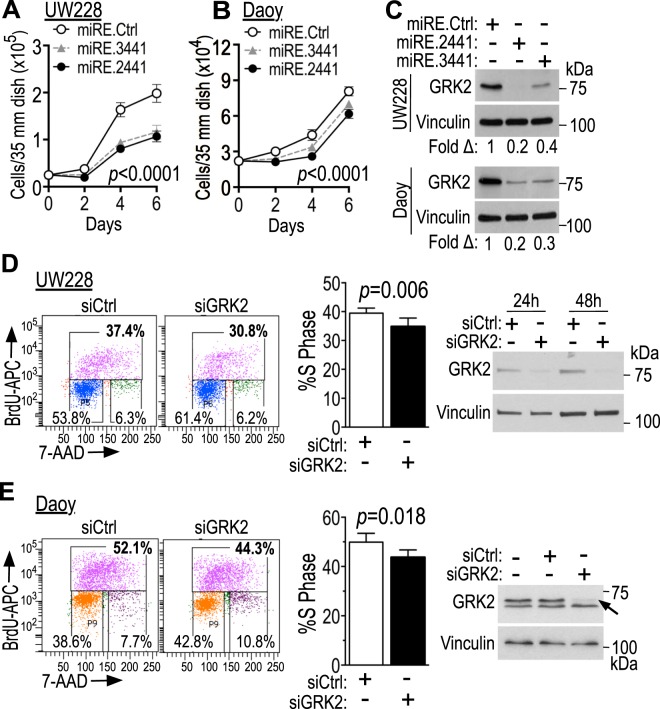
GRK2 knockdown slows growth and proliferation of UW228 and Daoy medulloblastoma cell lines. (**A–C**) Tva-expressing UW228 (**A**) and Daoy (**B**) cells were infected with human GRK2 knockdown miRE cassettes using the viruses pRDAV-mCherry-miRE.GRK2.2441 (miRE.2441), pRDAV-mCherry-miRE.GRK2.3441 (miRE.3441) or control pRDAV-mCherry-miRE.Ren.713 (miRE.Ren; miRE.Ctrl). They were then flow-sorted for mCherry-expressing cells, expanded for 8 days, re-seeded (2.2 × 10^4^ cells/35 mm dish UW228 and 2.5 × 10^4^ cells/35 mm dish Daoy) and grown for counting over 6 additional days in 10% FBS DMEM (UW228) or 1% FBS DMEM (Daoy). Live cells (cells excluding trypan blue) from three independent biological replicates (different passage cells, separate infections, different batch viruses) were counted manually every other day in each sample. Data points are means/SD of the three biological replicates. *P*-values were calculated over time using ordinary 2-way ANOVA with alpha 0.05, between cells infected with miRE.Ctrl *versus* miRE.GRK2.3441, and between cells infected with miRE.Ctrl *versus* miRE.GRK2.2441 (*p* < 0.001 for each pair over time). (**C**) GRK2 knockdown for A-B was validated by western blot using anti-GRK2 mouse monoclonal antibody (Santa Cruz cat #sc-13143) that detects a single GRK2 band. (**D**,**E**) UW228 (**D**) and Daoy (**E**) cells were transfected with 25 nM control or GRK2 siRNA mixed with 2.5 nM FAM-labelled siRNA and stained with apoBrdU-APC and 7AAD. FAM-positive cells were analyzed by flow cytometry 30 h after transfection. The left panels are representative flow cytometry curves of one of four independent experiments (**D**) and one of three (**E**) biological replicates, and right panels are western blots showing the level of GRK2 knockdown for cells shown in the flow cytometry curves. The anti-GRK2 antibody used in panel D is Santa Cruz cat#sc-13143 used in C. Panel E western blot used rabbit polyclonal, Santa Cruz cat #sc562, (1:6,000) that detected two bands here, of which the top one is GRK2 (arrow). Middle panels D-E show mean ± SEM of percent cells in S-phase in these experiments (n = 4 independent experiments for **D**, n = 3 biological replicates for (**E**), with *p*-values calculated using two-tailed unpaired t-tests.

We first examined the effect of GRK2 knockdown on growth of medulloblastoma cell lines UW228-2, Daoy and D283Med. Counting cells with GRK2 knockdown (miRE.3441 and miRE.2441) compared to control miRE (miRE.Ctrl with *Renilla* miRE) on day 2, 4 and 6 after plating revealed that the increase in cell number over time was slower when GRK2 was knocked down compared to control cells in both UW228 and Daoy cell lines, but not in D283Med (Fig. 1A–C and Suppl Fig. S1). BrdU uptake indicating cells in S-phase of the cell cycle was decreased in both UW228 and Daoy when GRK2 was knocked down with siRNA (Fig. 1D,E). For Daoy treated with scrambled si*Ctrl* 50.0% ± 2.1 (mean ± SEM) of the cells were in S-phase, while in si*GRK2* the S-phase comprised only 43.7% ± 1.7 of the cells, comprising a difference of 6.1% ± 0.8 between matched pairs in the three experiments. This constitutes a relative decrease of 0.12 ± 0.014-fold from baseline S-phase (1 to 0.877). In UW228 with scrambled si*Ctrl* S-phase comprised 39.5% ± 1.8 of the cells, while in si*GRK2* cells S-phase comprised only 34.8% ± 2.9, giving a difference in matched pairs of 4.7% ± 0.7 in the four experiments. This constitutes a relative decrease of 0.12 ± 0.02-fold from the baseline (1 to 0.88). The decreased S-phase following GRK2 knockdown in both cell lines indicates decrease in proliferation compared to controls (Fig. 1D,E). si*GRK2* knockdown did not affect protein level of its closest family member, GRK3 (Suppl Fig. S2), strengthening our conclusion that GRK2 contributes to growth and proliferation of medulloblastoma cell lines UW228 and Daoy.

### GRK2 overexpression attenuates, and knockdown enhances, cisplatin-induced apoptosis in medulloblastoma cell lines

**Figure 2:**
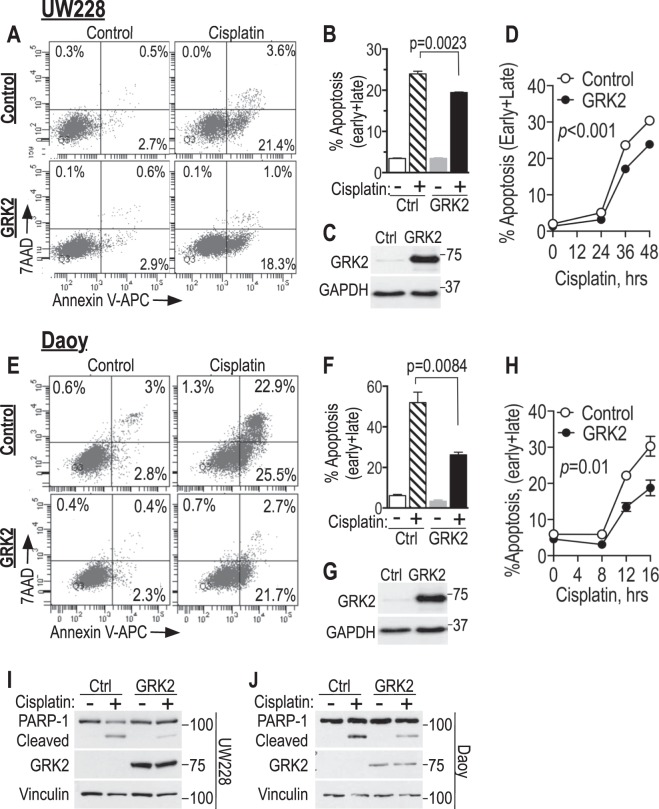
GRK2 overexpression diminishes cisplatin-induced apoptosis. Tva-expressing UW228 and Daoy cells were infected with pRDAV-mycGRK2-IRES-eGFP overexpressing human GRK2 or the control virus pRDAV-eGFP. Eight days later cells were plated overnight (2 × 10^5^ cells/well of 6-well plate) and then treated with cisplatin or vehicle. Apoptosis (AnnexinV/7AAD) was analyzed by flow cytometry in the eGFP-positive cells (**A**,**B**,**D–F**,**H**). (**A**,**B**) UW228 cells treated with 4 µg/ml cisplatin or vehicle control for 48 h. (**C**) Western blot of cells in A to assess GRK2 protein expression. (**D**) UW228 cells as in A,B, treated with cisplatin (4 µg/ml) for the times indicated. (**E**,**F**) Daoy cells, treated with 3 µg/ml cisplatin or control for 24 h. (**G**) Western blot of Daoy cells in F. (**H**) Daoy cells as in E,F treated with cisplatin (3 µg/ml) for the times indicated. Note that the range in panel H (4–16 h) is shorter than the time point measured in E,F (24 h). Data points and error bars in B,D,F and H are means ± SEM and represent early + late apoptosis (upper right + lower right quadrants, respectively) from three biological replicates for each of the cell lines tested (error bars are smaller than the symbols in **D**). Necrosis (upper left quadrants) was negligible. Similar results were seen in another set of experiments with 2 µg/ml cisplatin (not shown). (**I**,**J**) Cells infected as in A–H were flow-sorted for eGFP-positive cells following infection. Cells were expanded, treated with cisplatin or vehicle and cell lysates were assessed for PARP-1 cleavage by western blot: UW228 (**I**) were treated 24 h with cisplatin 4 μg/ml or vehicle. Daoy (**J**) were treated 8 h with cisplatin 3 μg/ml or vehicle. Statistical analysis: Bar graphs in panels B, D, F and H each are means ± SEM of three biological replicates. *p*-values in B and E were calculated using two-tailed unpaired student’s t-test. *p*-values in D and H were calculated using paired t-tests between eGFP and GRK2 samples for the different time points.

Cisplatin can induce apoptosis in medulloblastoma cells^60^ and is a mainstay of medulloblastoma therapy^61–64^. We therefore asked if GRK2 would protect medulloblastoma cells from cisplatin-induced apoptosis. We overexpressed GRK2 in UW228 and Daoy cells and assessed effect of cisplatin by AnnexinV/7AAD staining (flow cytometry) and PARP-1 cleavage (western blotting). Indeed, AnnexinV positivity and PARP-1 cleavage were both significantly attenuated in cells overexpressing GRK2 compared to controls (Figs 2 and S3). The effect was more pronounced in Daoy cells, where cisplatin induced more rapid and robust apoptosis compared to the UW228 cells (Fig. 2). In absence of chemotherapy, the low baseline apoptosis in both cell lines was not significantly affected by GRK2 overexpression. These data indicate that GRK2 overexpression can partially protect UW228 and Daoy cells from cisplatin-induced apoptosis.

**Figure 3:**
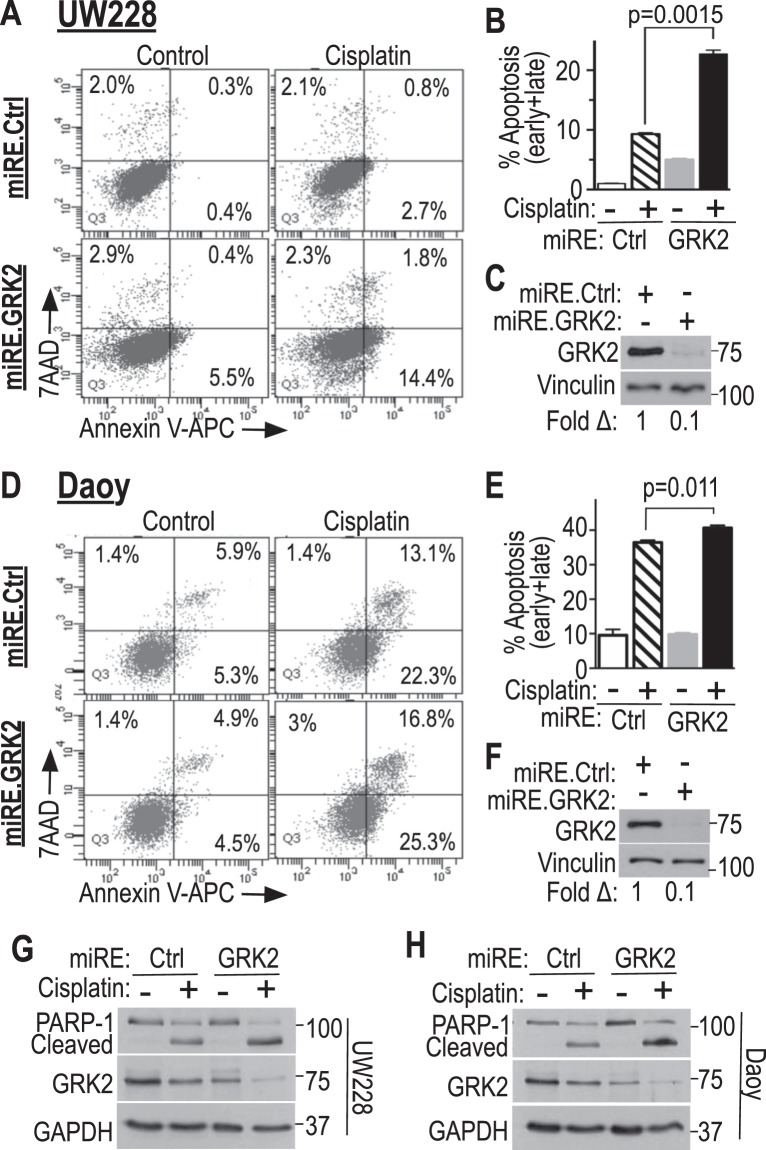
GRK2 knockdown augments cisplatin-induced apoptosis. Tva-expressing UW228 and Daoy cells were infected with human GRK2 knockdown viruses pRDAV-mCherry-miRE.GRK2.2441 (miRE.GRK2) or control pRDAV-mCherry-miRE.Ren.713 (miRE.Ctrl). Flow-sorted cells (mCherry-positive) were grown for 8 days after infection, re-plated overnight, treated with vehicle or cisplatin, and analyzed by flow cytometry for apoptosis (AnnexinV/7AAD) in the mCherry-expressing cells. (**A**,**B**) UW228 cells, plated overnight (2 × 10^5^/well in 6-well plates), treated with vehicle or 4 µg/ml cisplatin for 24 h then analyzed. (**D**,**E**) Daoy cells plated similarly, treated with vehicle or 3 µg/ml cisplatin for 24 h then analyzed. (**C**,**F**) Western blots showing GRK2 knockdown in UW228 cells (**C**) from A and in Daoy cells (**F**) from D. (**G**,**H**) Western blots to assess cisplatin-induced PARP-1 cleavage in UW228 (**G**) and Daoy (**H**) cells following GRK2 knockdown or control virus, with cisplatin or control treatments as in (**A**–**F**) except that duration of cisplatin treatment was 24 h for UW228 (**G**) and 16 h for Daoy (**H**). Shown: one experiment of three with similar results for each cell line. Statistics for panels B (two biological replicates) and E (three biological replicates): *p*-values were calculated using two-tailed unpaired student’s t-tests.

We then examined the reciprocal scenario of GRK2 knockdown followed by treatment with cisplatin using cells infected with RDAV virus carrying miRE.*GRK2* knockdown constructs compared to miRE.*Renilla* controls: cisplatin induced apoptosis (AnnexinV/7AAD) of control UW228 and Daoy cells, and this apoptosis was augmented in cells with GRK2 knockdown (Fig. 3). The increase in apoptosis by GRK2 knockdown was greater in UW228 cells, which were less sensitive to cisplatin at baseline compared to Daoy cells. Cisplatin-induced PARP-1 cleavage, also indicative of apoptosis, was increased by GRK2 knockdown compared to controls in both cell lines, solidifying the finding that GRK2 loss-of-function augments cisplatin-induced apoptosis (Fig. 3G,H). These data indicate that GRK2 partially protected UW228 and Daoy cells from the apoptotic effect of cisplatin, and that GRK2 loss rendered cisplatin more effective against these cells.

### GRK2 overexpression mitigates etoposide-induced apoptosis in medulloblastoma cells

**Figure 4:**
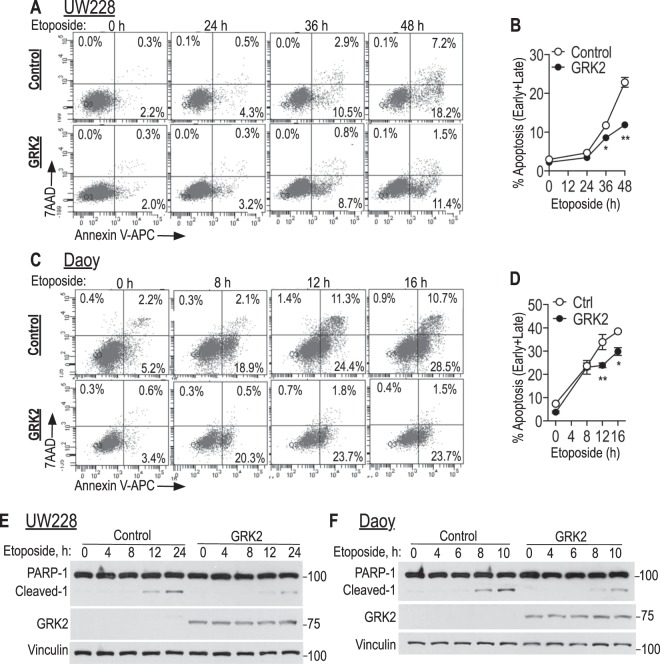
Etoposide-mediated apoptosis is mitigated in GRK2 overexpressing cells. (**A–D**) Tva-expressing UW228 (**A**,**B**) and Daoy cells (**C**,**D**) were infected with pRDAV-mycGRK2-IRES-eGFP virus to express human GRK2 or the control virus pRDAV-eGFP, grown for 8 days and then treated with etoposide (10 µg/ml for UW228, 8 µg/ml for Daoy) for the indicated times. Flow cytometry was used to assess apoptosis (AnnexinV/7AAD) in the eGFP-expressing cells. (**B**,**D**) are means ± SEM of early + late apoptosis of three biological replicates for each of these two cell lines; *p*-values: in B * represents *p* = 0.04 and ** represents *p* = 0.007; in D * represents *p* = 0.03 and ** represents *p* = 0.001, using unpaired student’s t-test. (**E**,**F**) Western blot for PARP1 cleavage in lysates of UW228 (**E**) or Daoy cells (**F**) infected with GRK2 or control vectors as in (**A**–**D**) that were treated with etoposide (10 µg/ml for UW228; 8 µg/ml for Daoy) for the indicated times. Supplemental Figure S4 provides a GRK2 western blot of cells used in (**E**,**F**) with longer exposure, to show native GRK2 level compared to overexpressed.

We then asked if the protective effect of GRK2 in medulloblastoma cells extended to treatment with etoposide, another chemotherapy that is commonly used against medulloblastomas^62,63^. As expected, treatment with etoposide induced apoptosis in both UW228 and Daoy cells (Fig. 4). Overexpression of GRK2 attenuated this apoptosis compared to eGFP controls when measured by flow cytometry (AnnexinV/7AAD) as well as by assessment of PARP-1 cleavage on western blot (Fig. 4 and Suppl Fig. S4). These data show that similar to cisplatin, increasing GRK2 expression conferred partial resistance to etoposide in these cell lines.

### GRK2-K220R and GRK2-S670A are similar to wildtype GRK2 in mitigating cisplatin-induced apoptosis in GRK2-knockdown cells

**Figure 5:**
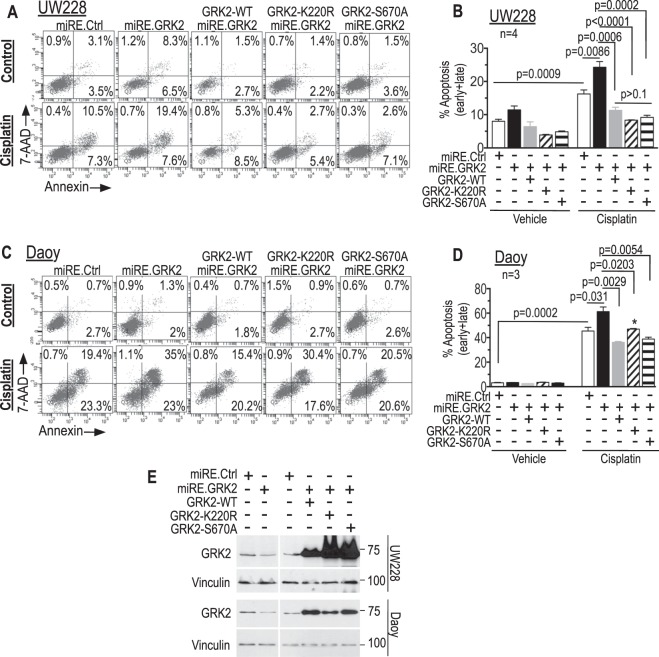
Kinase-inactive GRK2 retains ability to protect medulloblastoma cells from cisplatin-induced apoptosis. Tva-expressing cells were infected with virus that (1) knocks down endogenous GRK2, or (2) control vector using Renilla miRE, or (3) virus that simultaneously within the same vector knocks down endogenous GRK2 and instead expresses exogenous wild type GRK2, or GRK2-K220R (kinase dead), or GRK2-S670A (cannot be phosphorylated, is therefore constitutively active). The viruses were: (i) GRK2 knockdown (miRE.GRK2): pRDAV-eGFP-miRE.GRK2.2441; (ii) control (miRE.Ctrl): pRDAV-eGFP-miRE.Ren.713; (iii) endogenous GRK2 knockdown with exogenous wildtype GRK2 expression (GRK2-WT-miRE.GRK2): pRDAV-mycGRK2-IRES.eGFP-miRE.GRK2.2441; (iv) endogenous GRK2 knockdown with exogenous GRK2-K220R expression (GRK2-K220R-miRE.GRK2): pRDAV-mycGRK2.K220R-IRES-eGFP-miRE.GRK2.2441; (v) endogenous GRK2 knockdown with exogenous GRK2-S670A expression (GRK2-S670A-miRE.GRK2): pRDAV-mycGRK2.S670A-IRES-eGFP-miRE.GRK2.2441. Eight days after infection cells were re-plated overnight, treated with vehicle or cisplatin and eGFP-expressing cells were assessed for apoptosis (AnnexinV/7AAD). (**A**,**B**) UW228 cells treated with cisplatin 4 µg/ml for 48 h. (**C**,**D**) Daoy cells treated with cisplatin 3 µg/ml cisplatin for 24 h. (**E**) Western blot of unsorted cells from panels A and C. The miRE.GRK2 samples of both cell lines contained 80% eGFP positive cells and densitometry of the western blot showed 40% lower GRK2 (relative to vinculin) in the lanes of these cells compared to miRE.Ctrl lanes, indicating that in these experiments residual GRK2 protein in eGFP expressing miRE.GRK2 cells was approximately 50% that of miRE.Ctrl cells (i.e., 50% knockdown). All lanes are from the same blot at same exposure, with the right and left side of the blot having been moved to match the order of samples in panels B and D. Bar graphs show means ± SEM of four biological replicates in B and three biological replicates in D and represent the combined late + early apoptosis (upper and lower right quadrants, respectively). Necrosis (7AAD positive and AnnexinV negative) was minimal in all samples. *P-*values were calculated using unpaired two-tailed student’s t-tests (indicated in figure). *p = 0.0002 between GRK2-WT and GRK2-K220R (each with miRE.GRK2 knockdown).

We next examined if restoration of GRK2 expression can reverse the enhanced cisplatin-mediated apoptosis in GRK2 knocked-down cells. For this we used a vector that simultaneously expresses the miRE.GRK2 knockdown element directed towards the 3′UTR of GRK2 along with a myc-tagged wildtype GRK2 and compared it to the miRE.GRK2 knockdown vector or miRE.Renilla control in presence and absence of cisplatin. Figure 5 (black bars *versus* white bars in the cisplatin-treated cells in B and D) shows that knockdown of endogenous GRK2 augmented cisplatin-induced apoptosis in both UW228 and Daoy, similar to the findings in Fig. 3. Overexpression of wildtype GRK2 simultaneously with this knockdown of endogenous GRK2 by miRE.GRK2 reversed the augmented cisplatin-mediated apoptosis seen in miRE.GRK2-alone knocked down cells (Fig. 5B,D, grey *versus* black bars in the cisplatin-treated cells). These data indicate that sensitization of UW228 and Daoy cells to cisplatin-induced apoptosis following miRE.GRK2-mediated knockdown is specific to the decrease in GRK2 level, further solidifying the specificity of the functional effect of GRK2 knockdown in our system.

While GRK2-mediated effects are usually thought to require the kinase activity of GRK2^6,10,24–26,29–31,52^, GRK2 also has kinase-*in*dependent activities^52,65–69^. Such experiments typically use GRK2-K220R, a kinase dead GRK2 mutant that acts as a dominant negative in β2-Arrestin-mediated desensitization of GPCR signaling^65^ and/or GRK2-S670A, a mutant that cannot undergo the inhibitory S670 phosphorylation and thus has enhanced kinase activity towards GPCR substrates^2^. We therefore compared cisplatin-induced apoptosis in UW228 and Daoy in which we expressed the kinase-dead mutant GRK2-K220R or the phosphorylation-deficient GRK2-S670A to cells expressing wildtype GRK2. We overexpressed these mutants in vectors designed to simultaneously knock down the endogenous GRK2 by the miRE.GRK2 cassette, to diminish potential dampening that endogenous GRK2 may exert on effects of the GRK2 mutants. Both GRK2-K220R and GRK2-S670A were similar to wildtype GRK2 in their ability to mitigate the enhanced cisplatin-induced apoptosis seen in UW228 cells with knockdown of endogenous GRK2 (Fig. 5). Both mutants also protected Daoy cells, although GRK2-K220R seemed mildly less protective compared to wildtype GRK2 and GRK2-S670A (Fig. 5). These results show that GRK2 promotes partial resistance to cisplatin-induced apoptosis, and suggest that the GRK2-mediated protection from apoptosis is mostly via a pathway that is independent of the kinase activity of GRK2.

### GRK2 knockdown attenuates the increased AKT phosphorylation induced by cisplatin and etoposide

**Figure 6:**
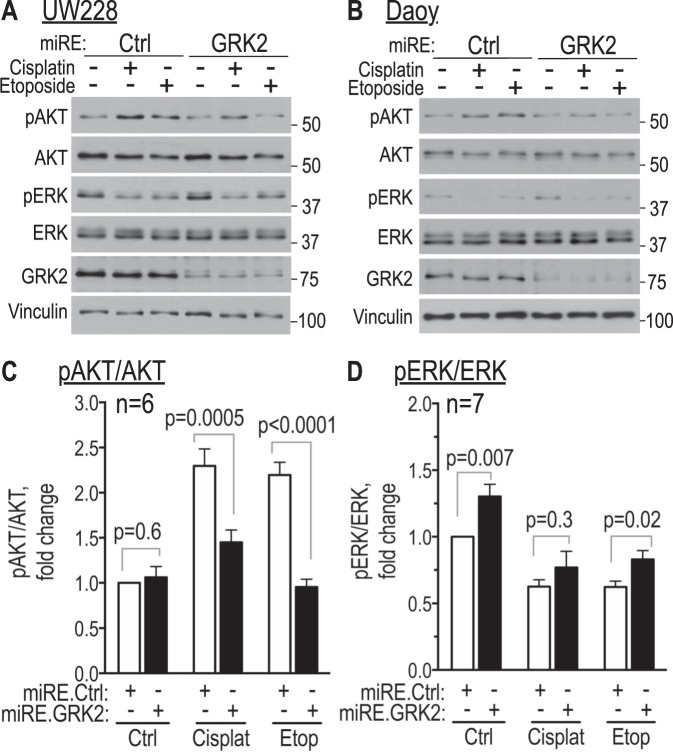
GRK2 knockdown mitigates the increase in AKT phosphorylation induced by cisplatin and etoposide. Tva-expressing UW228 (**A**) and Daoy (**B**) cells infected with GRK2 knockdown (miRE.GRK2) or control miRE.Ren (miRE.Ctrl) virus were flow-sorted for mCherry and expanded for up to two weeks. They were then seeded overnight and treated with cisplatin (UW228: 4 µg/ml, 24 h; Daoy: 3 µg/ml, 12 h) or etoposide (UW228: 10 µg/ml, 24 h; Daoy: 8 µg/ml, 12 h). Shown are western blots of whole cell lysates, representative of three experiments in UW228 and three (pAKT/AKT) or four (pERK/ERK) experiments in Daoy cells. Bar graphs represent means ± SEM of densitometry of pAKT/AKT (n = 6, combined three UW228 and three Daoy; **C**) and of densitometry of pERK/ERK (n = 7, combined three UW228 and four Daoy; **D**). GRK2 knockdown (miRE.GRK2, black bars); control miRE.Ren vector (miRE.Ctrl, white bars). Additional p-values beyond those in the panels are as follows: pAKT/AKT (panel C): for miRE.Ctrl, vehicle *versus* cisplatin *p* < 0.0001; for miRE.GRK2, vehicle *versus* cisplatin *p* = 0.06; for miRE.Ctrl, vehicle *versus* etoposide *p* < 0.0001; for miRE.GRK2 vehicle *versus* etoposide *p* = 0.5. pERK/ERK (panel D): for miRE.Ctrl, vehicle *versus* cisplatin *p* < 0.0001; for miRE.GRK2 vehicle *versus* cisplatin *p* = 0.004; for miRE.Ctrl vehicle *versus* etoposide *p* < 0.0001; for miRE.GRK2 vehicle *versus* etoposide p = 0.001. All comparisons are by two-tailed unpaired student’s t-test.

The PI3Kinase/AKT pathway plays a critical role in medulloblastoma biology and activation of the pathway in response to chemotherapy can contribute to chemoresistance^70–73^. Consistent with this, UW228 and Daoy control cells treated with cisplatin or etoposide both showed increase in phosphorylation of AKT-Ser473 (Fig. 6). Interestingly, GRK2 knockdown, which had no effect on baseline level of AKT phosphorylation, attenuated this chemotherapy-induced increase in AKT phosphorylation, leaving AKT phosphorylation at, or close-to, the levels seen in vehicle controls (Fig. 6). This is interesting in view of our finding that GRK2 knockdown enhanced apoptosis induced by cisplatin and etoposide (Figs 3 and 5).

ERK is another regulator of medulloblastoma growth whose activation in response to chemotherapy can also induce drug resistance^74,75^. Whereas phosphorylation of AKT-Ser473 was increased by cisplatin and etoposide, phosphorylation of ERK was diminished by both drugs (Fig. 6). Interestingly, GRK2 knockdown had no effect on this chemotherapy-induced decrease in ERK phosphorylation (Fig. 6). Taken together, these data show that GRK2 knockdown blocks the cisplatin- and etoposide-induced increase in AKT-Ser473 phosphorylation but does not affect the chemotherapy-induced decrease in ERK phosphorylation. This suggests that GRK2 may contribute to chemoresistance of medulloblastoma to cisplatin and etoposide via AKT.

## Discussion

From the small number of reports on the role of GRK2 in cancer cells it emerges that the effects vary and depend on the cancer type, promoting growth and survival in some^27,76–78^ and inhibiting it in others^79,80^. The work presented here provides evidence supporting a medulloblastoma-promoting effect of GRK2 by showing that decreasing GRK2 reduced growth and proliferation of UW228 and Daoy medulloblastoma cell lines. We further showed that GRK2 knockdown augmented, and GRK2 overexpression attenuated cisplatin- and etoposide-induced apoptosis in these cell lines. Furthermore, the protection by GRK2 was similar for wildtype GRK2 and for kinase-dead mutant GRK2-K220R, suggesting that the protection conferred by GRK2 was independent of the kinase activity of GRK2. Last, we showed that cisplatin and etoposide induced the phosphorylation of AKT-Ser473, and that knockdown of GRK2 blocked this AKT phosphorylation. This suggests that protection from etoposide- and cisplatin-induced apoptosis by GRK2 and augmentation of apoptosis by GRK2 knockdown may be mediated via AKT signaling. These findings suggest a role for GRK2 in promoting medulloblastoma growth and its resistance to chemotherapy.

GRK2 mRNA expression varies within each medulloblastoma subgroup, with overlap between groups (Fig. S5, derived from http://r2.amc.nl, R2: the Genomics Analysis and Visualization Platform)^81^. There is a trend towards shorter overall survival for patients with higher-than-median GRK2 mRNA in their medulloblastomas, but this difference is not statistically significant (Fig. S5A). These RNA data do not preclude a role for GRK2 *in vivo* in medulloblastomas, since GRK2 is also regulated at the protein level via proteasomal degradation^82–85^.

Approximately 25% of medulloblastomas belong to the SHH subgroup, characterized by aberrant SHH signaling. Smoothened, a GPCR-like receptor, is activated by SHH binding to the Smoothened inhibitor, Patched1 (PTCH1) and is important in cerebellar development and in medulloblastomas, whose origins are in the developing cerebellum^86,87^. The role of GRK2 in SHH/Smoothened signaling in invertebrate development and in mammalian cells^10,50–55,57,88^ suggest a potential link to medulloblastoma. GRK2 can directly interact-with and phosphorylate Smoothened following SHH stimulation^10^. Depending on conditions, GRK2 can have seemingly-opposite roles in this context: GRK2 is required for Smoothened signaling in some reports, while functioning in internalization and downregulation of signaling from Smoothened in others^10,50–52,54,57,59,88–90^. In this respect, the demonstration that GRK2 is required for SHH signaling in neural progenitor cells is interesting^59^. Also relevant, GRK2 is known to regulate CXCR4 signaling^91,92^. A link of GRK2 to medulloblastoma was hypothesized by Sengupta *et al*., who identified a subgroup of SHH medulloblastomas characterized by CXCR4 activation, showed that CXCR4 and SHH pathways functionally interact, and proposed that SHH may affect CXCR4 signaling via regulation of GRK2^93^. Another possible node for regulation of Smoothened signaling by GRK2 can be the SHH signaling negative regulator GPCR, GPR161, whose recruitment of β-arrestins and removal from the cilia are both regulated by GRK2^56^. Last, PTCH1, the receptor for SHH and negative regulator of Smoothened, directly interacts with cyclin B1 to suppress cyclin B1 nuclear accumulation and thus regulate cell proliferation^94^. GRK2 binds to PTCH1 through the GRK2 PTCH1-binding region to block PTCH1 binding to cyclin B1, thus relieving the inhibition on nuclear accumulation of cyclin B1 and promoting cell cycle progression^68^. We found a medulloblastoma-promoting effect of GRK2 in two SHH subgroup medulloblastoma cell lines, UW228 and Daoy^95–97^. The small number of SHH subgroup medulloblastoma cell lines that are available^95–97^ was limiting in terms of testing additional lines. Additionally, our experiments were unable to determine if the growth-promoting and protective roles of GRK2 were related to SHH pathway activation or independent of it. Limited experiments in D283Med cells (Group 3 medulloblastoma) with GRK2 knockdown over a six-day culture period did not reveal differences compared to control cells (Fig. S1). Considering the diverse signaling pathways affected by GRK2, it is possible that its action in medulloblastomas growth and protection from chemotherapy may be mediated via both Smoothened dependent and/or independent mechanisms.

In the majority of reports on GRK2 function, GRK2 desensitization of GPCRs typically requires GRK2 kinase activity. GRK2 kinase activity is maximal when GRK2 Ser670 is unphosphorylated, and is blocked with the kinase-dead GRK2-K220R^2,65^. There are a number of GRK2-mediated processes where the effect of kinase-dead GRK2-K220R is similar to the effect of wildtype GRK2, leading to the conclusion that these processes are independent of the GRK2 kinase activity. For example, in adipocytes GRK2-mediated endothelin-1-induced insulin resistance occurs by two mechanisms, one of which is a GRK2 kinase-independent inhibition of activation of Gαq/11^4^. For the thyrotropin receptor and the serotonin receptor 5-hydroxytryptamine_2C_, GRK2 N-terminal domain directly interacts with the activated Gαq and regulates its signaling in a GRK2 kinase-independent manner that is separate from desensitization of the receptor, which does require the kinase activity of GRK2^66^. Kinase-independent activity of GRK2 was also demonstrated for the histamine H2 receptor, a Gαs-coupled receptor, where receptor desensitization by GRK2 was independent of the GRK2 kinase activity, but receptor internalization and resensitization did required it^69^. Another example of differential GRK2 signaling is in astrocytes, where GRK2 modulates CCL2-mediated signaling to AKT in a GRK2 kinase-independent manner, while CCL2 signaling to ERK1/2 is modulated by GRK2 in a manner that does require the GRK2 kinase activity^67^. Last, the binding of GRK2 to PTCH1 blocks PTCH1 binding to cyclin B1 and relieves the PTCH1 inhibition on cyclin B1 nuclear accumulation: this GRK2 function is also independent of the kinase activity of GRK2, and can be achieved by kinase-dead mutant GRK2-K220R^68^. Here we reported that attenuation of cisplatin- and etoposide-induced apoptosis was similar in medulloblastoma cells expressing wildtype GRK2, the kinase-dead mutant GRK2-K220R^65^ or the non-phosphorylated constitutively-kinase-active mutant GRK2-S670A^2^. This suggests that GRK2-mediated protection of these medulloblastoma cells from cisplatin-induced apoptosis is at least partly independent of the kinase activity of GRK2 in a yet-unknown mechanism. Since GRK2 inhibitors being developed for cardiac protection^18–20,98^ are mostly focused on inhibiting kinase-dependent effects of GRK2, caution will be needed if they are considered for medulloblastoma.

The PI3K/AKT pathway plays an important pro-survival role in many cells, including in medulloblastomas^72,73,99–103^. Our data showed that cisplatin and etoposide increased AKT phosphorylation in both UW228 and Daoy cells. In cancer cells, an increase in AKT phosphorylation may act as a protective mechanism that can lead to chemoresistance^104,105^. Interestingly, knockdown of GRK2 diminished this cisplatin- and etoposide-induced increase in phospho-AKT compared to vehicle, suggesting that GRK2 mediates part of this chemotherapy-induced increase in AKT phosphorylation and may thus be a mediator of chemoresistance. The ERK pathway is hyperactive in some medulloblastoma tumors and feedback activation of it can contribute to reduced clinical efficacy of some chemotherapies^75,106^. Cisplatin and etoposide inhibited ERK phosphorylation in both UW228 and Daoy cells. Different than AKT, knocking down GRK2 did not alter ERK phosphorylation or its chemotherapy-induced decrease, indicating that GRK2 did not mediate this suppression of ERK phosphorylation or contribute to it. Of note, ERK is activated following β2-adrenergic receptor stimulation and phosphorylates GRK2 at S670, leading to inactivation of GRK2 kinase activity and constituting a regulatory feedback loop^2^. Taken together, our data indicate that GRK2 may mediate the increase in AKT phosphorylation induced by cisplatin and etoposide in UW228 and Daoy cells, potentially explaining the partial protection conferred by GRK2 against cisplatin- and etoposide-induced apoptosis. It will be interesting to test if blocking the GRK2-mediated cisplatin/etoposide-induced AKT phosphorylation will prevent emergence of resistance to these drugs.

In summary, we identified GRK2 as a contributor to regulation of cell growth and survival in two medulloblastoma cell lines. Decreasing GRK2 expression inhibited growth, diminished proliferation, and sensitized these cells to cisplatin-induced apoptosis, and increasing GRK2 expression mitigated cisplatin- and etoposide-induced apoptosis. GRK2-induced protection from cisplatin did not require the kinase activity of GRK2. Last, GRK2 mediated the chemotherapy-induced phosphorylation of AKT, suggesting that GRK2 may contribute to emergence of cisplatin resistance. Together, this is the first report of a growth-promoting function for GRK2 in medulloblastoma cells and its potential role in their response to chemotherapy.

## Material and Methods

### AnnexinV/7AAD apoptosis assay, BrdU proliferation assay

The APC AnnexinV kit (BD Pharmingen cat#550474) was used to assess apoptosis and the APC BrdU Flow Kit (BD Pharmingen cat#552598) was used to assess proliferation, both according to manufacturer’s instructions. Analyses were performed by flow cytometry focusing on the eGFP-expressing or FAM-labeled cells using an SLR II flow cytometer (BD Biosciences).

### Cell counting

Cells were mixed 1:1 with trypan blue in buffered isotonic salt solution. Cells were counted manually using a hemocytometer with cells excluding dye considered live and counted. Three aliquots from each sample were counted separately for each biological replicate.

### Cell culture

Cell lines used were UW228-2 (SHH medulloblastoma, from Dr. Silber)^107^, Daoy (SHH medulloblastoma, ATCC) and D283Med (Group 3 medulloblastoma, ATCC)^60^. Cell lines were authenticated using small tandem repeats: experiments were in cells frozen from tested aliquots. Cells were propagated in DMEM (Corning cat#MT10013CV) with 10% fetal bovine serum (FBS; Omega Scientific, cat#FB-01) unless stated otherwise.

### Plasmids

To express the genes and shRNAs below, viral vector plasmids were constructed in a modified version of pAlpha.SIN.SF.EGFP.wPRE^108^ (gift of Dr. A. Schambach, Hannover Medical School, Germany) that we term pRDAV (manuscript in preparation). These plasmids were used to produce virus stocks for overexpression or knockdown of GRK2 in human cell lines. Human GRK2 knockdown was achieved using the miR-E (miRE) backbone^109^ combined with shRNAs designed by the SplashRNA algorithm^110^; this miRE combination was included in the 3′ region of the indicated viral vectors. shRNA sequences and partial miRE sequences were synthesized as 97-mers (Supplementary Table S1), amplified and cloned into a miRE backbone as described^109^. Initial pRDAV knockdowns were performed using one of two miRE-containing plasmids: pRDAV-mCherry-miRE.GRK2.2441 or pRDAV-mCherry-miRE.GRK2.3441; the negative control miRE plasmid (pRDAV-mCherry-miRE.Ren.713, abbreviated as miRE.Ctrl or miRE.Ren) contained the published miRE for *Renilla* luciferase^109,110^. A vector expressing an N-terminally MYC-tagged human GRK2 (pRDAV-mycGRK2-IRES-eGFP) was used to overexpress GRK2 and was compared to a negative control (pRDAV-eGFP). For experiments with mutant GRK2 we simultaneously knocked down endogenous GRK2 using miRE.GRK2.2441, which is directed to the 3′UTR of endogenous GRK2, and overexpressed wildtype or mutant GRK2 from the same vector in the following plasmid constructs: pRDAV-mycGRK2-IRES-eGFP-miRE.GRK2.2441, pRDAV-mycGRK2.K220R-IRES-eGFP-miRE.GRK2.2441, and pRDAV-mycGRK2.S670A-IRES-eGFP-miRE.GRK2.2441. Control vectors for these mutant GRK2 experiments were pRDAV-eGFP-miRE.GRK2.2441 (GRK2 knockdown) and pRDAV-eGFP-miRE.Ren.713 (*Renilla* knockdown, negative control).

### Transfections and virus infections

To enhance biosafety we used a modified RCAS-TVA system^111,112^ for introducing genes and shRNAs into cultured cells by viral vector infection. Cells were first engineered to stably express TVA, the receptor for avian retroviral vectors displaying subgroup A envelope of avian sarcoma-leukosis virus (ASLV). Tva was amplified from the BarTeL transgene we produced previously^113^. Cells were infected with RDAV-Puro-IRES-Tva coated with the VSV-G envelope followed by puromycin selection (1 µg/ml) of pooled cells. Virus was generated by co-transfection of HEK-293T cells with the pRDAV plasmids along with plasmids for *gag*/*pol* and for *env*, and the filtered (0.45 µ) supernatants (days 2–5 post-transfection) were used for infection. All experiments, other than those to produce the TVA-expressing cell lines, used the ASLV subgroup A envelope to produce virus. To overexpress or knock down GRK2, the Tva-expressing cells were infected with the pRDAV avian virus expressing the desired constructs described under Plasmids. For viral infections, 10^5^ cells/well were seeded in wells of a 6-well plate and when approaching 40% confluence were infected with virus. After 24 h media containing virus were replaced with regular growth medium and cells were grown for 8 days with regular feedings and splitting to obtain sufficient cells for the experiment.

For siRNA transfections, 2 × 10^5^ cells/6-well were seeded in regular growth medium. On the following day the medium was replaced with Opti-MEM reduced serum medium (ThermoFisher Scientific cat#31985070) and cells were transfected with 25 nM of a mix of four GRK2-specific siRNAs (Santa Cruz Biotechnology cat#sc-29337) or with control siRNA (Qiagen cat#1027281) along with 2.5 nM FAM-labelled (ThermoFisher Scientific cat#AM4620) siRNA as marker, using 5 µl Lipofectamine 2000 (ThermoFisher Scientific cat#11668019) for 7 h. After incubation, siRNA containing medium was replaced with regular growth medium for 23 h and desired experiment was performed.

### Reagents

Cisplatin (Accord Health care Inc., stock 1 mg/ml) and etoposide (Nova Plus, stock 20 mg/ml) were from the pharmacy at Children’s Hospital Los Angeles. GRK2 siRNA (pool of 4 GRK2-specific siRNAs, cat#sc-29337) was from Santa Cruz Biotechnology. Non-silencing negative control siRNA (cat#1027281) was from Qiagen, AllStars. FAM-labeled siRNA negative control (cat#AM4620) was from Life Technologies. Other reagents are listed in specific procedures. All other reagents are from Sigma-Aldrich Co unless otherwise specified.

### Western blotting

Cell lysates were prepared in RIPA buffer comprising of 50 mM Tris-HCl, pH 8.0, 150 mM sodium chloride, 1.0% Igepal CA-630 (NP-40), 0.5% sodium deoxycholate, 0.1% sodium dodecyl sulfate, 50 mM NaF, with freshly added 10 μM PAO, 2 mM heated (95 °C × 5 min) sodium ortho-vanadate, 1 mM PMSF and protease inhibitor cocktail (Thermo Fisher Scientific catalog #A32955). Proteins were resolved on 10% or on 8–12% gradient SDS-PAGE gels. Transfer, Enhanced Chemiluminescence and western blotting were performed as described^114^. Antibodies used were: anti-GRK2 rabbit polyclonal antibody (cat#sc-562, 1:6,000), anti-GRK2 mouse monoclonal antibody (cat#sc-13143, 1:3,000), anti-GAPDH mouse monoclonal antibody (cat#sc-32233, 1:10,000), anti-vinculin mouse monoclonal antibody (cat#sc-25336, 1:6,000), anti-pERK mouse monoclonal antibody (cat#sc-7383, 1:3,000) and anti-ERK1 rabbit monoclonal antibody (cat#sc-94, 1:6,000) from Santa Cruz Biotechnology. Anti-PARP-1 rabbit monoclonal antibody (cat# 9542, 1:3,000), anti-pAKT(Ser473) mouse monoclonal antibody (cat#4051, 1:3,000) and anti-AKT rabbit monoclonal antibody (cat#9272, 1:6,000) were from Cell Signaling Technology. Secondary antibodies were HRP goat anti-rabbit IgG (cat#PI-1,000 to 1:2,000) and HRP horse anti-mouse IgG (cat#PI-1000, 1:2,000) from Vector Laboratories. Primary antibody incubations were at 4 °C and secondary antibodies at room temperature for 1 hour. Blots were scanned into pdf files, opened in Photoshop, and exposure level was adjusted equally across the whole blot (lighter by 0.3–0.75). Densitometry was performed using Image Studio Lite software (LI-COR Biosciences) or ImageJ 1.5.0.i to analyze the scanned blots.

### Statistics

Data is expressed as mean ± SEM or SD derived from independent experiments and/or independent biological replicates in each of the cell lines as stated in the Legends. Biological replicates differed in terms of being performed independently and/or in a separate experiment, in several places by different lab members. They also differed in the Tva-expressing cells used, that had been cultured separately for differing periods, were infected independently using virus that was produced separately by different lab members and/or harvested on a different day. Data were analyzed using GraphPad Prism version 6.0 h and version 7.0 d for Mac (www.graphpad.com). Method for determining significance is listed in figure legends. p < 0.05 was considered statistically significant.

## Supplementary information

Supplementary Figures and Legends
