# Supplementary material for: GRK2 promotes growth of medulloblastoma cells and protects them from chemotherapy-induced apoptosis
Source: Sci Rep. 2019 Sep 25;9:13902. doi: 10.1038/s41598-019-50157-5 (PMC6761358; doi:10.1038/s41598-019-50157-5)
Supplement: Supplementary file 1 — Supplementary Figures and Legends [file 41598_2019_50157_MOESM1_ESM.pdf]

GRK2 promotes growth of medulloblastoma cells and protects them from chemotherapy-induced apoptosis

Anup S. Pathania, Xiuhai Ren, Min Y. Mahdi, Gregory M. Shackleford, and Anat Erdreich-Epstein

SUPPLEMENTARY FIGURES and LEGENDS

Figure S1

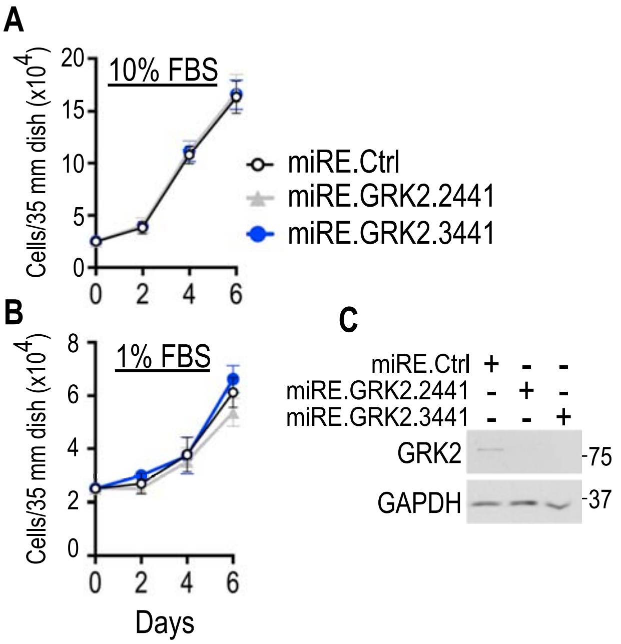

Figure S1. GRK2 knockdown does not affect growth of D283Med cells

Tva-expressing D283Med medulloblastoma cells were infected with human GRK2 knockdown viruses pRDAV-mCherry-miRE.GRK2.2441 (miRE.GRK2.2441), pRDAV-mCherry-miRE.GRK2.3441 (miRE. GRK2.3441) or control pRDAV-mCherry-miRE.Ren.713 (miRE.Ctrl). Eight days after infection 2.5×10^4 cells/well/35 mm dish were seeded in 10% FBS DMEM. The following day the medium was replaced with either fresh 10% FBS DMEM (**A**) or 1% FBS DMEM (**B**). Live cells (cells excluding trypan blue) were counted on days 2, 4, and 6. Shown are means±SD of counts from three experiments. **C)** D283Med cell lysates demonstrating knockdown of GRK2 protein by the miRE.GRK2 constructs.

Figure S2. si*GRK2* knockdown diminishes GRK2 protein level but does not affect the level of GRK3 protein.

Daoy cells seeded at 10^5/well in 6-well plates were transfected with *GRK2* siRNA 30nM or 60nM (Santa Cruz #SC29337) or control siRNA (Qiagen #1027281) for 24 hrs. Whole cell lysates were resolved on 10% SDS-PAGE. Western blotting reveals efficient knockdown of GRK2 by the si*GRK2* but not by control siRNA and no change in GRK3 protein (nor in vinculin or GAPDH).

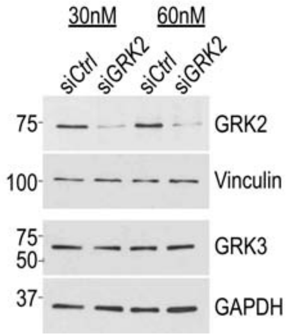

Figure S3. GRK2 overexpression protects UW228 medulloblastoma cells from apoptosis induced by increasing doses of cisplatin.

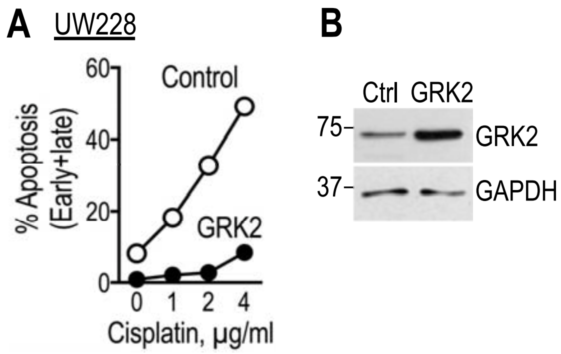

A) UW228-Tva cells infected as in Figure 2 and treated for 48 h with increasing concentrations of cisplatin or vehicle. Apoptosis was analyzed in the eGFP-expressing cells by flow cytometry for AnnexinV/7AAD. Shown are single flow cytometry samples per time point. **B**) Western blot verification of GRK2 expression of samples used in A. Densitometry shows that GRK2 overexpression increased GRK2 protein level by 3.4 fold in this experiment.

Figure S4. GRK2 overexpression in Daoy and UW228 cells for Figure 4E and 4F

Western blot of lysates from cells used in Figure 4E and 4F overexpressing GRK2 or vector control using longer exposure of the blots to allow assessment of the extent of overexpression of GRK2.

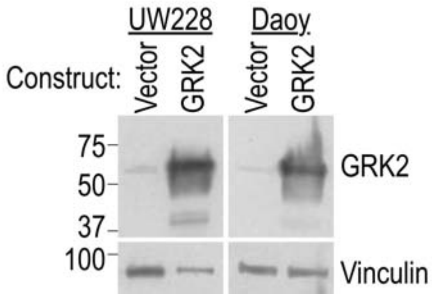

Figure S5

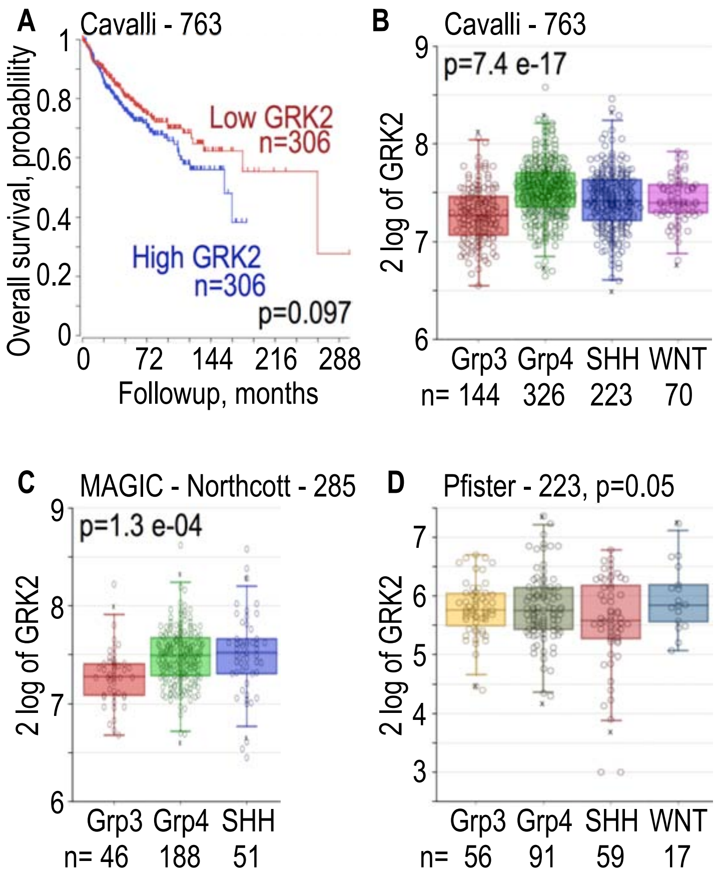

Figure S5. GRK2 mRNA is expressed in medulloblastomas

Interrogation of the R2: Genomics Analysis and Visualization Platform (http://r2.amc.nl) for GRK2 (ADRBK1, adrenergic, beta, receptor kinase 1). **A-B)** Analysis of the dataset Tumor Medulloblastoma – Cavalli - 763 - rma_sketch - hugene11t. Kaplan-Meier curve shows overall survival of patients whose tumors had GRK2 mRNA above (blue) or below (red) the median. **C)** Analysis of the dataset Tumor Medulloblastoma MAGIC - Northcott - 285 - rma-sketch - hugene11t. **D)** Tumor Medulloblastoma – Pfister - 223 - MAS5.0 - u133p2.

ORIGINAL DATA OF WESTERN BLOTS IN FIGURES 1-6:

Original for Fig 1C UW228

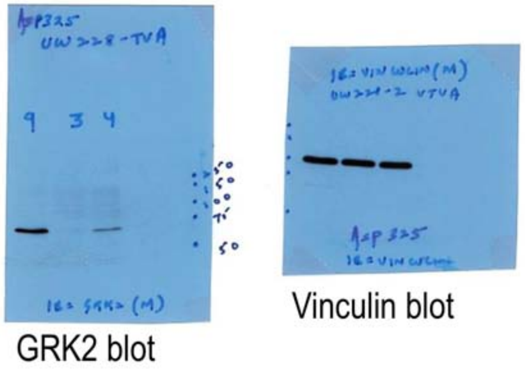

Original for Fig 1C Daoy

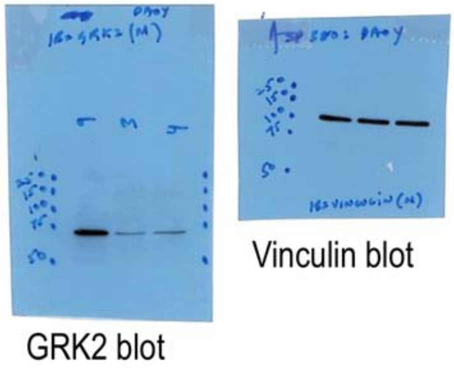

Original for Fig 1D UW228

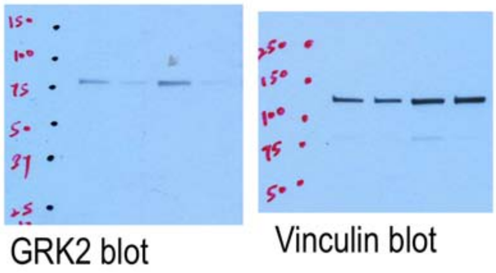

Original for Fig 1E Daoy

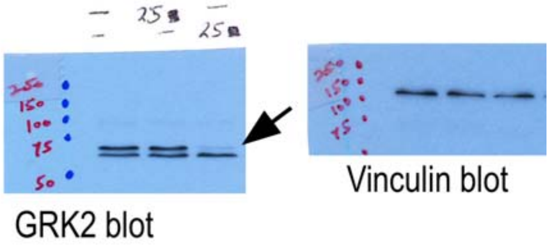

Original blots for Figure 2C

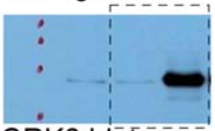

GRK2 blot

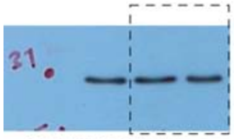

GAPDH blot

Original blots for Figure 2G

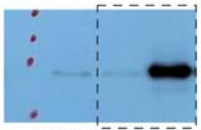

GRK2 blot

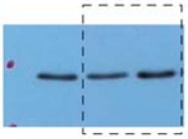

GAPDH blot

Original blots for Figure 2I

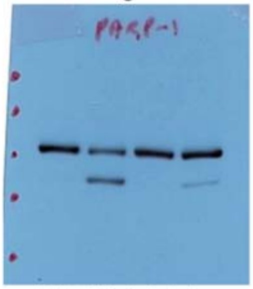

PARP-1 blot

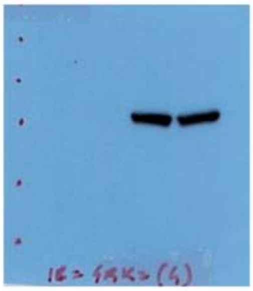

GRK2 blot

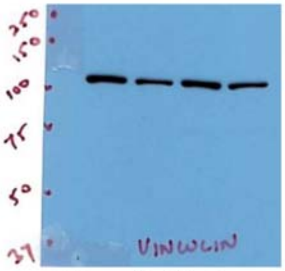

Vinculin blot

Original blots for Figure 2J

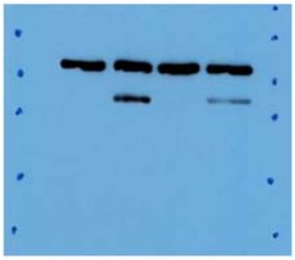

PARP-1 blot

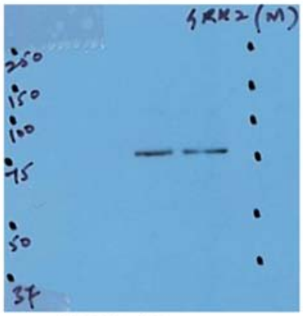

GRK2 blot

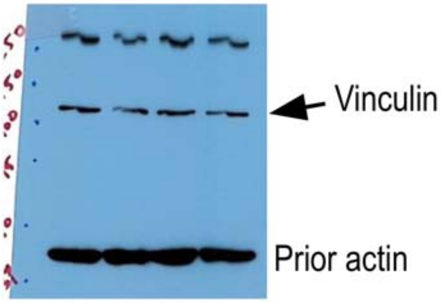

Vinculin blot

Original for Figure 3C

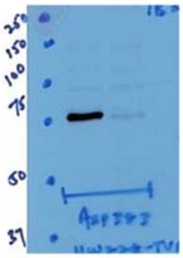

GRK2 blot

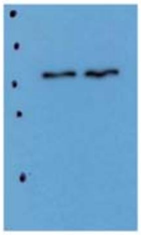

Vinculin blot

Original for Figure 3F

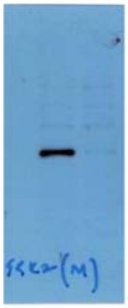

GRK2 blot

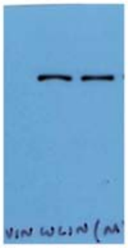

Vinculin blot

Original for Figure 3G

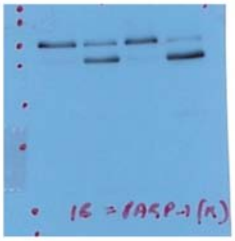

PARP-1 blot

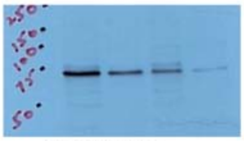

GRK2 blot

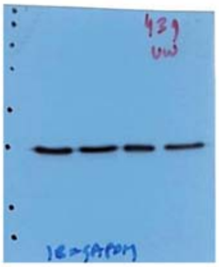

GAPDH blot

Original for Figure 3H

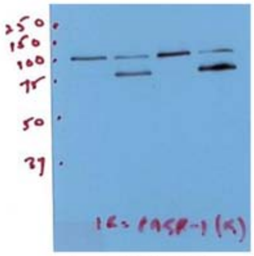

PARP-1 blot

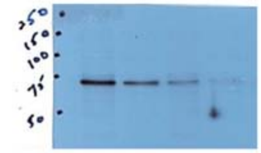

GRK2 blot

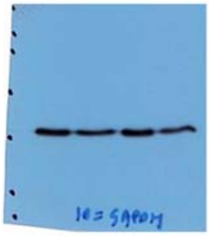

GAPDH blot

Original blot for Figure 4G

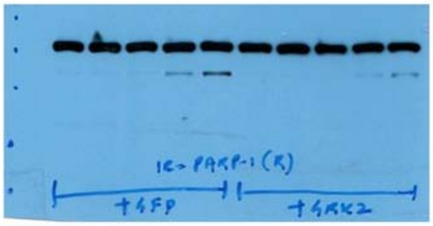

PARP-1 blot

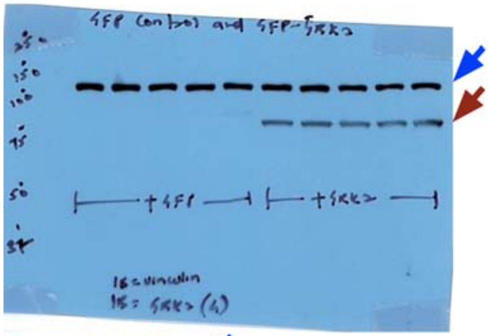

Vinculin blot

GRK2 blot

Orignal blot for Figure 4H

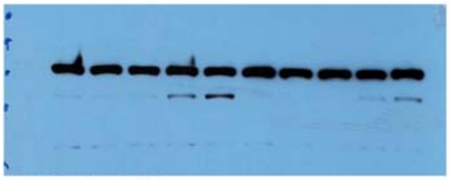

PARP-1 blot

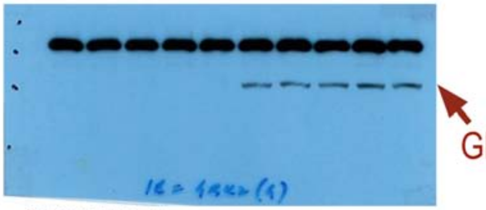

GRK2

GRK2 blot

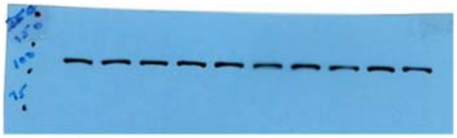

Vinculin blot

Originals for Figure 5E-UW228

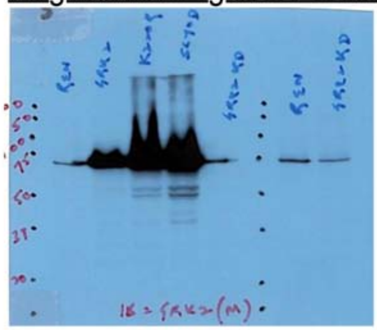

GRK2 blot:

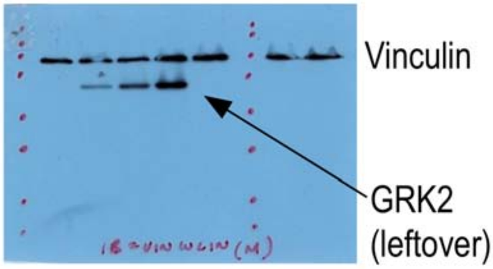

Vinculin

GRK2 (leftover)

Vinculin blot (after GRK2 blot)

To arrange the bar graphs and blots in a rational manner, the right-most two lanes of the blot (Ren/miRE.Ctrl and GRK2 knockdown/miRE.GRK2) were placed on the left side of panel 5E. The extra knockdown lane (KD) lane was not included in 5E as it was a duplicate and had spillage from the intensely-positive adjacent S670 lane.

Originals for Figure 5E-Daoy

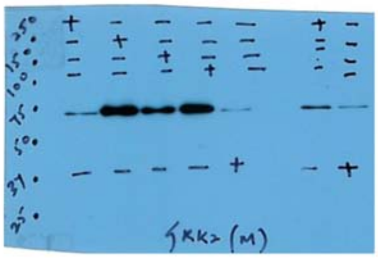

GRK2 blot

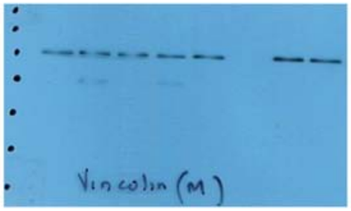

Vinculin blot (after GRK2 blot)

Original blots for Figure 6A

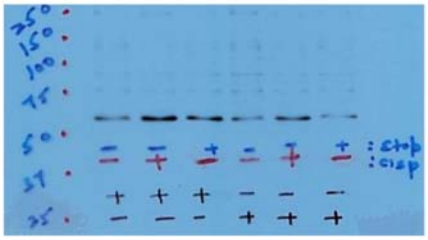

p~AKT(Ser473) blot

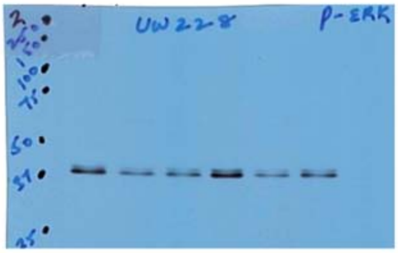

p~ERK blot

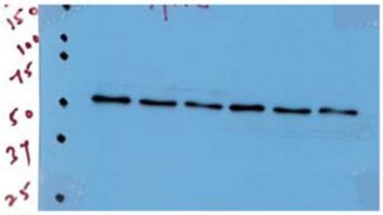

Total AKT blot

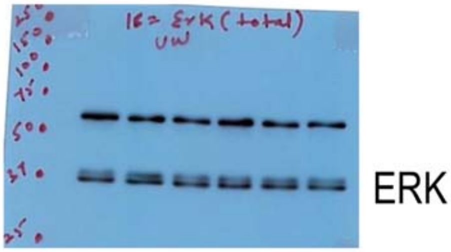

Total ERK blot (after AKT blot)

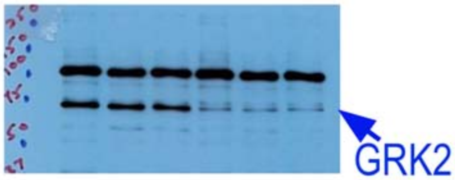

GRK2 blot (after vinculin)

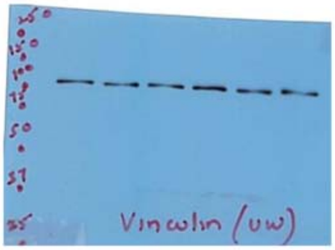

Vinculin blot

<u>Original blots for Figure 6B</u>

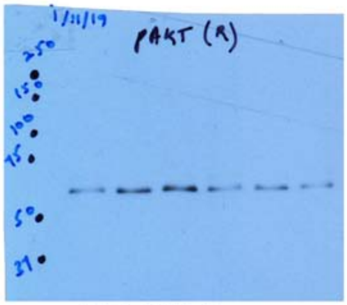

p~AKT blot

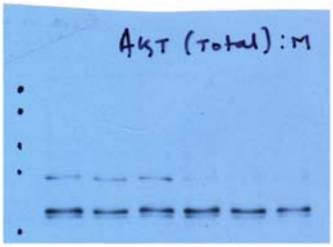

AKT

AKT blot (after GRK2 blot)

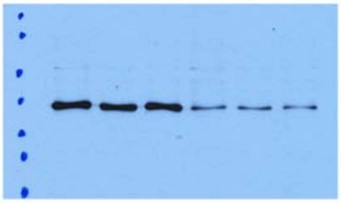

GRK2 blot

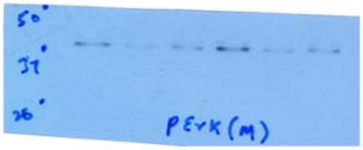

p~ERK blot

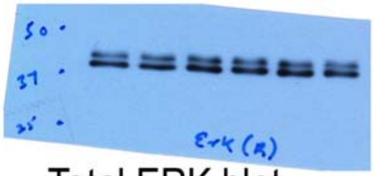

Total ERK blot

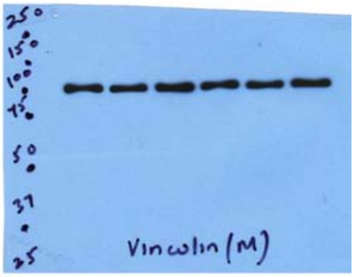

Vinculin blot
